# Secondary Raw Materials from Residual Carbon Fiber-Reinforced Composites by An Upgraded Pyrolysis Process

**DOI:** 10.3390/polym13193408

**Published:** 2021-10-04

**Authors:** Alexander Lopez-Urionabarrenechea, Naia Gastelu, Alberto Jiménez-Suárez, Silvia G. Prolongo, Adriana Serras-Malillos, Esther Acha, Blanca María Caballero

**Affiliations:** 1 Chemical and Environmental Engineering Department, Faculty of Engineering of Bilbao, University of the Basque Country (UPV/EHU), Plaza Ingeniero Torres Quevedo 1, 48013 Bilbao, Spain; naia.gastelu@gmail.com (N.G.); adriana.serras@ehu.eus (A.S.-M.); esther.acha@ehu.eus (E.A.); blancamaria.caballero@ehu.eus (B.M.C.); 2 Materials Science and Engineering Area, ESCET, University Rey Juan Carlos, C/Tulipán s/n, 28933 Móstoles, Spain; alberto.jimenez.suarez@urjc.es (A.J.-S.); silvia.gonzalez@urjc.es (S.G.P.)

**Keywords:** carbon fiber-reinforced polymers, secondary raw materials, carbon fiber, recycling, pyrolysis, hydrogen

## Abstract

This paper presents a process where carbon fibers and hydrogen can be recovered simultaneously through a two-stage thermal treatment of an epoxy-carbon fiber composite. For this purpose, some pieces of epoxy resin reinforced with carbon fiber fabrics have been fabricated and, after curing, have been pyrolyzed in an installation consisting of two reactors. In the first one, the thermal decomposition of the resin takes place, and in the second one, the gases and vapors coming from the first reactor are thermally treated. Once this process is completed, the solid generated is oxidized with air to eliminate the resin residues and carbonaceous products from the fibers surface. The recovered carbon fiber fabrics have been reused to make new cured parts and their electrical and mechanical properties have been measured. The results show that it is possible to obtain carbon fiber fabrics that can be processed as they leave the recycling process and that retain 80% of the tensile modulus, 70% of the flexural strength, and 50% of the interlaminar shear strength. At the same time, a gaseous stream with more than 66% by volume of hydrogen can be obtained, reaching a maximum of 81.7%.

## 1. Introduction 

The demand for carbon fibers continues to grow at an unstoppable rate. The global carbon fiber market is expected to surpass 300 kt production by 2027, which supposes a 11.7 % compound annual growth rate (CAGR) for the 2020–2027 period [1]. Such is the growing use of this material, that the industry itself is beginning to warn that, in the near future, the demand will exceed the supply capacity [2]. Carbon fiber is used almost entirely as reinforcement in plastic matrix composites (mainly epoxy-type), better known as carbon fibers reinforced plastics/polymers (CFRP). CFRP offers a ratio between weight and mechanical properties that is practically unattainable for many traditional materials, so their immersion in the aviation, automotive and large structures construction industries is relentless, in addition to many other applications [3]. Besides, these types of materials are extending their use to new applications that might benefit from their lower density, such as structural composite based batteries [4], supercapacitors [5], and are also being adapted to new emerging manufacturing technologies, such as 3D printing [6]. 

The extensive use of carbon fiber/epoxy composites has led during last years to the seek for new opportunities regarding their modification to obtain new functionalities apart from the structural one: manufacturing modification to improve quality [7], reducing their cost [8], increase versatility, and consider more sustainable processing and recycling possibilities. This last aspect has also led to research in new polymer degradable and reprocessible epoxy matrices used to impregnate carbon fibers [9], although their still present important drawbacks such as limited mechanical properties. These drawbacks motivate further research in the field and alternatives in recycling conventional epoxy carbon fiber composites as the present work shows. There is currently no legislation affecting CFRP waste, beyond Directive 2000/53/EC on End of Life Vehicles if CFRP are part of a vehicle. This means that the waste generated in the production and end-of-life phases of this type of material lacks a specific collection and treatment system other than landfill or incineration. In other words, there is no obligation or target for recycling. While the environmental authorities are waking up, it seems that the industry itself is beginning to worry about this problem. The question is simple: there may be carbon fiber supply problems in the future, and at the same time, we are discarding or destroying the materials that contain it. Not only that, but there is also evidence (even at commercial scale) that carbon fiber can be recovered using less energy than for its production, generating a lower cost product that retains its mechanical properties to a degree that allows it to be reused in not all, but many current applications [2,10,11,12,13]. These aspects, together with a global concern about the need of evolving towards a circular economy concept, makes the need of looking for new ways of recycling and reusing these composites a priority for the research community [14,15]. 

The reclamation of carbon fibers requires the removal of the polymeric resin that embeds the fibers, which, as a rule, is a thermoset. There are multiple ways addressed to deal with the removal of the thermoset resin from the CFRP waste, commonly divided into mechanical, thermal and chemical methods [16,17,18]. The strengths and weaknesses of each of the techniques are described in detail in the specialized literature, but among them, it appears that pyrolysis-based technologies are the most mature technology for CFRP recycling [19,20,21]. Through this process, carbon fibers have been recovered and subsequently used for a variety of applications, e.g., use in cars [22] or boats [23], and reinforcement for thermoplastics such as PP [24] or 3D printing [25]. However, apart from constraints related to the creation of new markets for recycled CFRP, recent studies indicate that efforts should still be made to achieve high quality materials in CFRP recycling processes [26]. The first one concerns the morphology of the carbon fibers and the products manufactured with them (fabrics, yarns, etc.). One of the easiest ways to reuse the fibers is cutting or crushing them. This approach also allows reusing them as fillers without removing completely the resin surrounding the fiber. When the shortening of fibers is avoided, they can be obtained as fluffy fiber mats with random orientation and, less commonly, in the same product as they were originally. This last approach will be addressed in this paper; it will allow using them in the same way as the original, that is, with the same orientation, and avoiding further processing of products made of fibers. The second one has to do with the possibility of recovering materials other than carbon fibers. The authors have been studying, for several years, the possibility of recovering some high-value substances, namely hydrogen, from the thermal decomposition products of the polymeric resin. Such an investigation has been done by applying a thermal treatment [27,28] or a thermal-catalytic treatment [29,30] to the gases and vapors evolved during resin decomposition. As far as the authors are concerned, there is no research precedent for attempting to recover substances other than carbon fibers in thermoset-based CFRP pyrolysis processes; in fact, the development of the method for the production of hydrogen simultaneously with the recovery of carbon fibers has been recognized by the Spanish and European patent offices [31]. In this sense, this article is itself a novelty in the field of carbon fiber composite materials recycling. In addition, this work presents two main novelties with respect to previously published papers by the authors: for the first time, the gas and vapor treatment process is applied to a cured part, and new CFRP parts are manufactured from recycled fibers. 

## 2. Materials and Methods 

### 2.1. Components and Manufacturing of Virgin CFRP and Recycled CFRP

The CFRP pieces used in this study consisted of 10 carbon fiber fabrics in an epoxy system. The carbon fiber fabric (0/90 type) was provided by Hexcel (Parla, Spain), and its main properties are shown in Table 1. AS4C is a PAN-based carbon fiber usually used for industrial purposes, with high performance requirements, such as aerospace industry. 

The epoxy system, widely used in structural applications in the aeronautical industry, consisted of Araldite LY 556 acting as precursor (based on bisphenol A diglycidyl ether) and XB 3473 as hardener, both supplied by Huntsman (Salt Lake City, UT, USA). The properties of these two products can be seen in Table 2, together with the properties of the mixture concretely used in this case. As can be observed, Araldite LY 556 and XB 3473 make a very low-viscosity mixture, very suitable for infusion-based manufacturing processes. 

**Figure 1:**
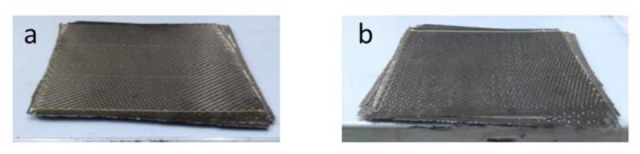
Appearance of the manufactured vCFRP1 (**a**) and vCFRP2 (**b**).

Using these materials, two CFRP pieces were manufactured by vacuum-assisted resin infusion molding (VARIM), followed by a 140 °C/8 h curing process. The resulting virgin CFRP pieces (vCFRP) are presented in Figure 1. Subsequently, the carbon fiber fabrics recovered from vCFRP1 and vCFRP2 were used to manufacture other two pieces, this time based on recycled CFRP (rCFRP), also by using the same epoxy system and manufacturing process. The main characteristics of the vCFRP and rCFRP pieces are summarized in Table 3. 

### 2.2. Carbon Fiber Recovery Process

**Figure 2:**
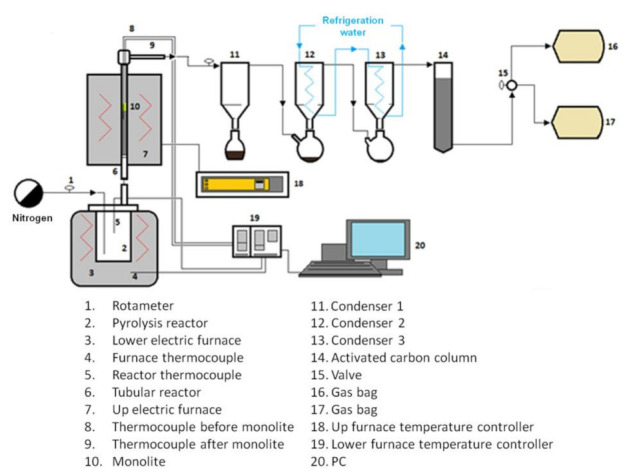
Flowsheet of the lab-scale installation used for recovering the carbon fibers (note that, in this case, no nitrogen was used in the pyrolysis step).

In order to recover the carbon fibers, the vCFRP samples were subjected to a thermal process consisting of pyrolysis, followed by controlled oxidation. This way, the epoxy resin was decomposed by pyrolysis and then air was used to remove the remaining carbonaceous substance covering the fibers (char). The carbon fiber recovery process took place in a lab-scale installation composed of a stainless steel 3.5 L tank reactor in series with a stainless-steel tubular reactor (2.54 cm diameter, 50 cm length) filled with a solid bed consisting of 0.5–1 mm particles of high-alumina refractory material. The outlet of the tubular reactor was connected to a cooling, vapor condensation and gas cleaning section, consisting of 3 condensers and an activated carbon column. In the experiments, the tank reactor was heated up to 500 °C at 3 °C min^−1^ in the absence of any carrier gas. Due to increasing temperature, the epoxy resin cracked into gases and vapors that passed through the tubular reactor, where they were thermally treated in order to reduce the organic content of the pyrolysis liquids and to maximize the hydrogen content in gases [29,30]. When resin decomposition finished, air was fed to the tank reactor to start the oxidation step. Oxidation took place also at 500 °C, which is the optimum temperature in order to remove the char while the lowest damage to the carbon fibers occur [28,32]. The amount of air needed for oxidation was calculated, assuming that the weight difference between the solid obtained after pyrolysis and the carbon fiber content in the CFRP corresponded to the amount of char generated, which was considered to be 100% carbon. In order to study the influence of the airflow rate, a low flow super-stoichiometric oxidation (O1) and a high flow stoichiometric oxidation (O2) were used. A summary of the experimental conditions used in the carbon fiber recovery process is presented in Table 4. On the other hand, a lab-scale installation can be seen in Figure 2. 

### 2.3. Analytical Techniques

#### 2.3.1. Analysis of Liquid and Gaseous Products

The composition of pyrolysis gases was determined by chromatographic analysis coupled with thermal conductivity and flame ionization detectors (GC-TCD/FID, Agilent 7890A, CA, USA). A gas standard provided by Air Liquide and consisting of H_2_, CO, CO_2_, CH_4_ and alkanes and alkenes up to C6 was used to identify and quantify the gas components. The higher heating value (HHV) of the gases was calculated from the HHV of the individual components found in the gas mixture. The composition of the liquids was established by gas chromatography (GC, Agilent 6890, CA, USA) coupled to mass spectrometry (MS, Agilent 5973, CA, USA). In this case, the chromatograph was not calibrated and therefore the composition is given in % area. Such chemicals identified by the MS search engine with a quality value < 85% were classified as “not identified”. 

#### 2.3.2. Characterization of Carbon Fibers

A Hitachi S-3400N microscope (Tokyo, Japan) was used for the morphological characterization of the carbon fibers by scanning electron microscopy (SEM). The accelerating voltage was set to 10 kV and, mainly, a secondary electron detector was used. In that way, samples were analyzed at ×1000 and ×2000 augmentations. Longitudinal and cross-sectional images were used in order to determine the carbon fibers’ diameter. Besides, carbon fiber surface roughness and the presence of sizing and/or polymeric residues were also established. Apart from SEM, X-ray diffraction (XRD) was used to determine the crystallinity of the virgin and recycled carbon fibers, by using the Panalytical X’Pert PRO θ/2θ diffractometer (Malvern, UK).

#### 2.3.3. Characterization of vCFRP and rCFRP

The electrical conductivities of both the virgin and recycled CFRP pieces were measured following the ASTM D257 standard test method, by using the Keithley 2410 instrument (Ohio, United States), which is able to set a specific voltage (V) to the studied material and measure the electrical current (I) passing through it. Each sample was tested for five different voltages between 0 and 0.05 V. The mechanical properties (Young modulus, flexural strength and interlaminar shear strength, ILSS) were measured following the ASTM D3039, D790 and D2344 respectively, employing the Zwick Z-100 universal testing machine (Ulm, Germany), which is characterized by a three-point bend fixture, where the distance between supports (span) can be adjusted as established in the standard and a 5 kN load cell. Samples used were 12 mm with and 80 mm long in order to ensure. For tensile strength determination, 15 mm width and 100 mm long test samples were prepared by using the Struers (Copenhagen, Denmark) Labotom 3 cut-off machine (cut-off wheel 10S25) and then, tensile test was performed using a 100 kN load cell at 1.3 mm min^−1^ velocity. Flexural strength was determined on a 12 mm width and 80 mm long test tube (cut with the Struers machine) by using a 5 kN load cell and 1.0 mm min^−1^ velocity. Concerning ILSS, the width and length of the test tubes are defined related to the thickness, therefore width = 2 × thickness and length = 6 × thickness. Span value was set to 4 × sample thickness and velocity to 1 mm·min^−1^.

## 3. Results and Discussion

### 3.1. Recovery of Carbon Fibers

Table 5 shows the yields of the solid, condensate and gas fractions obtained in the pyrolysis step of the vCFRP pieces. The temperatures used in the two reactors during the pyrolysis step are included in brackets after P1 and P2 in the table. Solid and condensate yields were calculated by weight difference. In the first case, the difference was between the weight of the initial sample and that of the solid obtained after pyrolysis. In the case of condensates, the weight difference was measured in the reactors, condensers and pipelines of the installation. For this reason, the yield of condensates is split into “collected liquids”-those collected in the condensers, and “other condensates”-such liquids remaining in the reactors and pipelines after the experiment. Gas yield was calculated by difference. Table 5 also shows the composition of the collected liquids and the dry composition and HHV of the pyrolysis gases. The gas composition is given free of nitrogenous, oxygenated and sulfur compounds. At last, the quantity of the remaining solid after the oxidation step is also included in Table 5. This value is given in percentage with respect to the initial vCFRP mass. The operating parameters that differentiate the two oxidation stages (time, airflow and excess air, respectively) are included in parentheses after O1 and O2. 

The yields shown in Table 5 show that, in both cases, the main product obtained was the solid fraction, which constituted 72.6% by weight in the case of vCFRP1 and 75.9% by weight in the case of vCFRP2. Based on the previous experience of the authors and other research teams, it can be said that this solid fraction included the carbon fibers (65.8 wt.% in vCFRP1 and 67.6 wt.% in vCFRP2) and a mixture of char and non-decomposed polymeric resin [29,30,33,34]. Considering that the operating conditions of the tank reactor were the same in both experiments, the slight difference between the two solid yields must be a consequence of (1) the different fiber content of the two pieces and (2) the deviation of the results that usually occurs in this type of experiments. As far as condensate and gas yields are concerned, there was significant variation between the two experiments. In experiment P1, 23.4 wt.% of condensates and 4 wt.% of gases were generated, while in experiment P2, the condensate yield was only 14.2 wt.%, so the gaseous fraction increased to almost 10 wt.%. The decrease in the yield of the collected liquids (from 8.9 to 1.7 wt.%) is particularly remarkable, since it is the liquid product collected in the systems used for this purpose (condensers), as opposed to the other condensates, whose yield may be higher or lower depending on the specific characteristics of the installation. The increase in the gaseous fraction and the decrease in the liquid fraction were direct consequences of the temperature used in the tubular reactor in each of the tests. At P1, the temperature was 700 °C, while at P2, it was 900 °C. A higher temperature in the reactor that treated the vapors and gases from the resin decomposition caused more cracking reactions and higher conversions. This provoked the breakdown of long molecules that may end up forming part of the condensates when cooled, giving rise to smaller molecules that remained in gaseous phase at room temperature. The authors have observed similar behavior in a previous work on pyrolysis of CFRP containing epoxy resin [29].

The gas obtained in both cases was composed exclusively of hydrogen, carbon monoxide and methane, presenting, in both cases, a percentage by volume of hydrogen greater than 65%. This is a very important result, since hydrogen percentages higher than 50% by volume in a gas mixture allow the economically profitable separation of hydrogen by pressure swing adsorption (PSA), in order to be used in high value applications. In fact, this is the main objective of using the authors’ patented gas and vapor treatment method, because it involves the production of a secondary raw material derived from the polymeric resin [31]. It was quite surprising that the amount of CO_2_ observed in the gases was in trace amounts, which may mean that one of the routes of hydrogen generation in both experiments was the dry reforming of hydrocarbons. Additionally, as can be seen in Table 5, hydrogen production was higher in the experiment performed at 700 °C (81.7 vs. 66.4 vol.%), which could be a consequence of the reverse water gas shift reaction, which is favored by high temperatures and would also produce more CO. It is also possible that methanation reactions occurred in P2 experiment starting from previously formed H_2_, as has been reported by the authors in biomass pyrolysis processes [35]. However, the complete explanation about gas composition cannot be addressed without studying the composition of the condensates. 

In this respect, the composition of the collected liquids was very different between the two experiments. In P1, a liquid composed mainly of phenol and derivatives (> 70% area), together with aromatic hydrocarbons, was generated. However, in experiment P2, a mostly aqueous liquid was obtained (> 80% area), where phenol and its derivatives did not reach 10% in area, and aromatic hydrocarbons disappeared. The production of this kind of very aqueous liquids in the thermal treatment of CFRP has been exclusively reported by the authors up to now, and it is another desirable consequence of the vapor and gas treatment method [28]. The most likely pathway for the removal of organic compounds in liquids is cracking, which, as discussed above, is favored by the high temperatures used in P2. Most of the organic compounds in the liquid phase were oxygenated (phenol and derivatives), which may explain the formation of H_2_O as a product in liquids when cracking. In addition, if the reactions mentioned previously as responsible for the decrease of H_2_ in gases (reverse water gas shift reaction and methanation) occurred, both could be sources of H_2_O. In summary, it can be said that the author’s patented method for treating vapors and gases coming from the decomposition of waste CFRP can be successfully applied to cured, epoxy-based CFRP pieces. This leads to a gas fraction very rich in hydrogen and, at the same time, to a minority aqueous liquid fraction, with a very low content of phenol and derivatives.

Concerning the oxidation step, and as commented on in the Materials and Methods section, two different oxidation processes were applied (O1 & O2) in order to analyze the influence of the airflow rate in the reactor. O1 represents a low-flow oxidation process, since the amount of air needed was fed in 120 min long. On the other hand, in O2, air amount was fed in 30 min. As it can be seen in Table 5, the sample recovered from vCFRP1, which will be named rCF1, showed less mass than the fiber quantity of vCFRP1 –54.7% vs 65.8%, see Table 3. On the other hand, the sample recovered from vCFRP2 (from now on rCF2) presented higher weight than the fiber quantity of vCFRP2—69.3% vs 67.6%, see Table 3—(all in wt.%). Thus, char and resin removal efficiency would equal 133.4% for O1 and 95.8% for O2. That is, it is clear that in O1 experiment, apart from char and resin, some carbon fiber content was also oxidized, which can happen when increasing temperature or time of oxidation [36]. On the other hand, in O2 experiment, there was not a loss of carbon fiber content, and char and resin were almost completely removed. These results do not agree with those obtained by the authors in a previous work about the oxidation of pyrolyzed CFRP [28]. On that occasion, the optimum operating parameters for the oxidation step were 500 °C, 120 min and 1.8 L min^−1^ of air (55% excess). On the contrary, in this work, under very similar operating conditions (even less excess), the oxidation of the carbon fibers happened. The explanation may lie in two main reasons. On the one hand, the epoxy resin was different in this work, leading to a different decomposition mechanism and perhaps to the formation of less char and/or of a more volatile nature. On the other hand, the presence of a single piece of CFRP in the reactor in this work was likely to improve the solid–gas contact, so that oxidation occurred more efficiently than in the previous experiments, where a strip of pre-preg more than one meter long was wound on itself inside the reactor. Consequently, an over-performance took place in this case for very similar oxidation conditions. 

**Figure 3:**
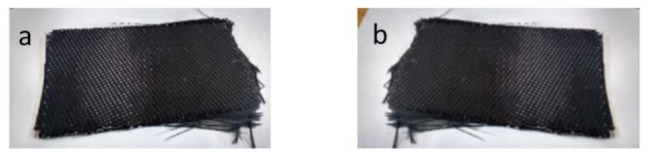
Carbon fiber fabrics after pyrolysis-oxidation process: rCF1 (**a**) and rCF2 (**b**).

A picture of both rCF pieces is shown in Figure 3. In both cases, the rCF fabrics maintained the original shape and structure, so it was possible to directly use them for remanufacturing without any kind of post-treatment. The maintenance of shape and structure has been also reported in other works concerning CFRP pyrolysis [37,38] and it constitutes an important property in terms of primary energy demand for the remanufacturing process, as addressed by the investigation of He et al. [20]. However, in spite of this shape conservation, it could be observed with the naked eye that the fabrics were more oxidized on the lower part of them, which was the part closest to the air feeding point in the reactor (the CFRP pieces were placed standing upright in the reactor, parallel to the airflow). For this reason, each of the fabrics was divided into two before characterization: rCF1-down, rCF1-up, rCF2-down and rCF2-up. Samples with the suffix “down” refers to the sample side placed on the bottom part of the reactor, from where the air is fed during the oxidation step. By contrast, the sample with the suffix “up” refers to the sample side placed on the upper part of the reactor.

**Figure 4:**
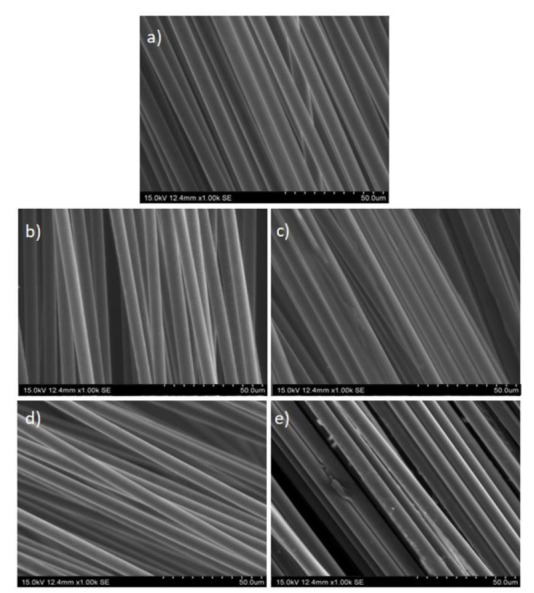
SEM images of the longitudinal view of carbon fibers: (**a**) Virgin carbon fibers (vCF); (**b**) Recovered carbon fibers in O1 located on the bottom part of the reactor (rCF1-down); (**c**) Recovered carbon fibers in O1 located on the upper part of the reactor (rCF1-up); (**d**) Recovered carbon fibers in O2 located on the bottom part of the reactor (rCF2-down); (**e**) Recovered carbon fibers in O2 located on the upper part of the reactor (rCF2-up).

Figure 4 shows a 1000-fold augmentation of the longitudinal SEM view of the recovered carbon fibers. Additionally, the same view of virgin carbon fibers-named vCF-has also been included in the figure in order to compare and contrast the differences and similarities between them. Concerning the bottom parts of the fabrics-(b) rCF1-down and (d) rCF2-down-their appearance is quite similar to the virgin carbon fibers in (a), without any sign of surface roughness and attachment between fibers. Consequently, it could be concluded that resin removal efficiency was good, and clean carbon fibers were obtained in these cases. Nonetheless, based on results in Table 5, it could be reasonably thought that sample rCF1-down lost certain quantity of carbon fibers; therefore, sample rCF2-down probably presented the best recovery conditions. By contrast, some residues could be appreciated in figures (c) and (e)-the upper parts of the fabrics, rCF1-up and rCF2-up, respectively. If both are compared, it seems that, char removal efficiency in (c) was higher than in (e). This could be explained by the fact that the amount of air is smaller in O2 oxidation (that corresponding to rCF2), and at the same time, the linear velocity of the fed air into the reactor is higher in this experiment, so the contact time between air and sample is smaller this time.

**Figure 5:**
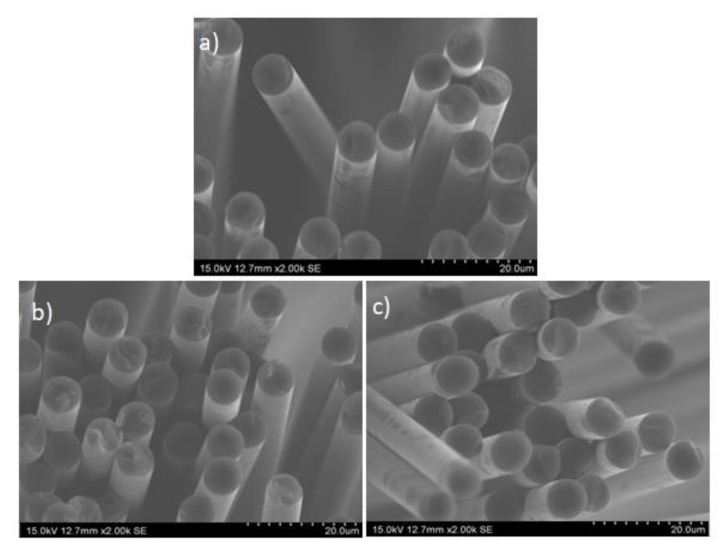
SEM images of the cross-sectional view of the studied carbon fibers: (**a**) Virgin carbon fibers (vCF); (**b**) Recovered carbon fibers in O1 experiment located on the bottom part of the reactor (rCF1-down); (**c**) Recovered carbon fibers in O2 experiment located on the bottom part of the reactor (rCF2-down).

In view of the SEM images presented in Figure 4, it can be said that the regions of the fabrics with the cleanest fibers were those corresponding to the bottom part of the reactor (“down”). However, as this is the region in greatest contact with air, it was necessary to check whether the fibers had also been partially oxidized. For this to be possible, the cross-section diameter of the fibers more likely to be damaged was measured (rCF1-down and rCF2-down). Figure 5 compiles the cross-sectional SEM images of these two samples with a 2000-fold augmentation in comparison to that of the virgin carbon fibers as a reference scenario. As it can be seen in Figure 5, this case, the possible damage suffered during the carbon fibers recovery process, was not appreciable for the naked eye. Therefore, the cross-sectional diameter of the three samples-vCF, rCF1-down, rCF2-down-was repeatedly measured (×10 each), so as to result in an average diameter for each of the studied samples. The results are presented in Table 6, where it can be seen that the average cross-sectional diameter of the virgin carbon fibers is 7.25 μm, while recovered carbon fibers showed a slight reduction in comparison: 7.06 μm for rCF1-down and 7.15 μm for rCF2-down. The biggest cross-sectional diameter reduction appears in the carbon fibers recovered after O1 experiment-rCF1-down-, which had the longest oxidation time. Indeed, in the O1 experiment, fed air had smaller linear velocity, what means that fed air and sample CFRP1, inside the reactor, were longer in touch. Nonetheless, the highest diameter reduction, compared to the virgin carbon fibers, amounted to 2.6%. In fact, if the calculated errors related to the average cross-sectional measured diameters are taken into consideration, it could be assumed that there are no significant differences between the virgin and the recovered carbon fibers cross-sectional diameters. In conclusion, it could be stated that carbon fibers were not significantly damaged during the recovery process in terms of morphology. These results agree with those obtained by other authors under similar oxidation conditions [39,40]. 

**Figure 6:**
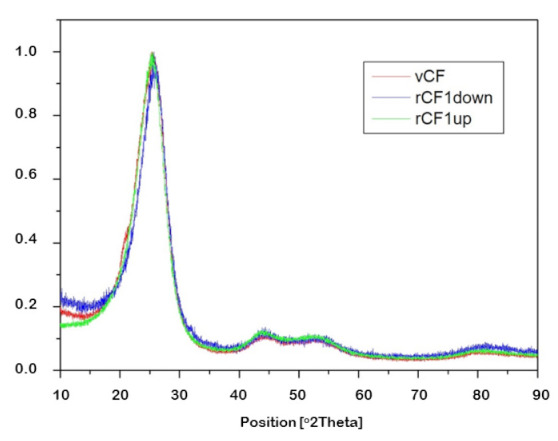
X-ray diffractogram of virgin carbon fibers (vCF) and recovered carbon fibers in O1 experiment (rCF1-up and rCF1-down).

**Figure 7:**
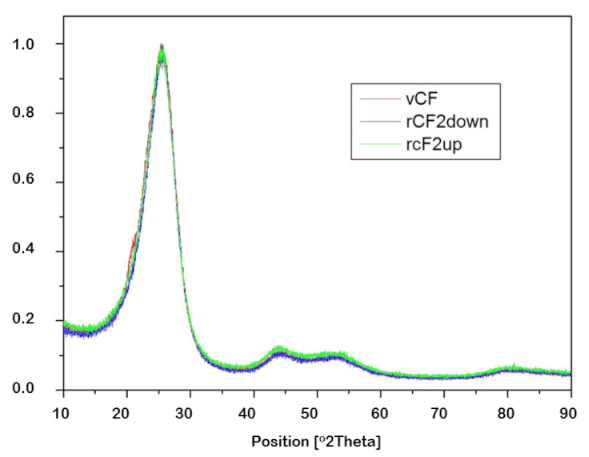
X-ray diffractogram of virgin carbon fibers (vCF) and recovered carbon fibers in O2 experiment (rCF2-down and rCF2-up).

The crystallinity of the samples was analyzed by X-ray diffraction. In this particular case, the interest laid in determining if the recovery process generated a loss of crystallinity in the recovered carbon fibers. For that, diffraction peaks of the virgin carbon fiber sample (vCF) and each of the recovered carbon fiber samples-rCF1-down, rCF1-up, rCF2-down and rCF2-up-were analyzed, aided by the software X’Pert High Score Plus, so as to quantify the full width at half maximum (FWHM) and the value of the diffraction plane (2θ) in each case. Figure 6 shows diffraction peaks of the recovered carbon fibers in O1 experiment, in addition to the virgin carbon fibers’ peak so as to ease the comparison between them. Likewise, Figure 7 shows diffraction peaks of the recovered carbon fibers in O2 experiment and the diffraction peak of the virgin carbon fibers. Finally, Table 7 compiles the values of FWHM and 2θ for each of the carbon fiber samples mentioned.

Two characteristics regions from the XRD can be observed in Figure 6 and Figure 7, the one regarding parameters of the (002) plane at 2θ angles around 25.5° and the one regarding the plane (101) at 2θ angles around 43.5°. Once again, no significant differences were observed among the virgin carbon fiber and the reclaimed ones, which implies that the recycling process did not affect the crystallinity of the carbon fibers. 

### 3.2. Characterization and Performance of Recycled CFRP

#### 3.2.1. Electrical Conductivity 

**Figure 8:**
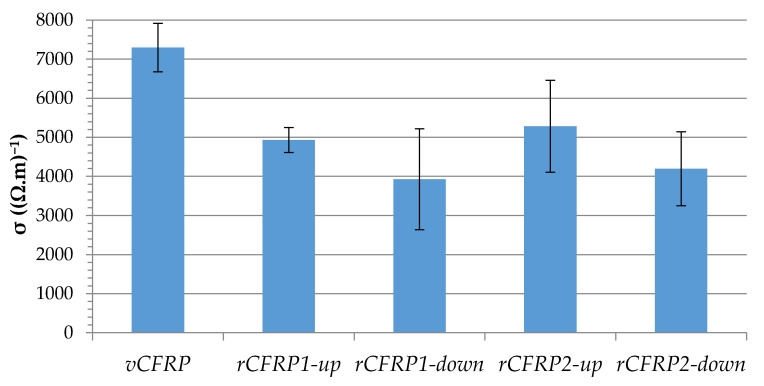
Electrical conductivity of recycled CFRP parts in comparison to the mean value of virgin CFRP pieces.

Electrical conductivity was used as an indirect measure of the quality of the recovered carbon fibers. Taking into account that the epoxy resin is an insulating material, a decrease in the electrical conductivity of the CFRP provides information related to the unproper transmission of electricity through the carbon fiber fabric, which could indicate some kind of damage, break or alterations in the material. For this to be possible, the four pieces of recovered carbon fiber fabrics were used to manufacture recycled CFRP parts, and named rCFRP1-up, rCFRP1-down, rCFRP2-up, rCFRP2-down. The electrical conductivity of the four rCFRP pieces was measured and compared to that of virgin CFRP, specifically, the mean electrical conductivity of vCFRP1 and vCFRP2, named as vCFRP. It must be taken into consideration that vCFRP is not a real piece; it was not fabricated, it only represents the main value of vCFRP1 and vCFRP2. The results are plotted in Figure 8, where the mean value of two measures and their error bar are presented. 

Figure 8 clearly illustrates that recycled composite materials suffered a decrease in the electrical conductivity values, ranging between a loss of 27.6% for sample rCFRP2-up and 46.2% for rCFRP1-down. It is worth noting that the two parts of the fabrics that were in the area of the reactor close to the air inlet were the ones that generated the CFRPs with the lowest electrical conductivity (rCFR1-down and r-CFRP2-down), while the CFRPs made from the fabrics that were farthest away from air contact were the ones with the highest conductivities (rCFRP1-up and rCFRP2-up). Comparing the fibers obtained from experiment O1 and experiment O2, it can be seen that the fibers from experiment O1, which had a longer oxidation time and excess air, appeared to be more damaged in both the upper and lower parts. These results confirm the oxidation of the fibers that could already be assumed in view of the remaining weight of solid obtained in this test and shown in Table 5. On the other hand, the best results were obtained with the fibers recovered in the upper part of the vCFRP2, oxidized during less time, and with smaller air quantity. The diameter reduction of carbon fibers has been previously reported as a sign of carbon fiber damage which afterwards leads to reduction of electrical conductivity of the composites reinforced with these fibers. Park et al. used short carbon fibers to reinforce phenolic matrices, and they found that treatments at higher temperatures lead to a reduction in carbon fiber diameters followed by a consequent reduction in electrical conductivity capabilities of the reinforced composites [41].

#### 3.2.2. Mechanical Properties 

**Figure 9:**
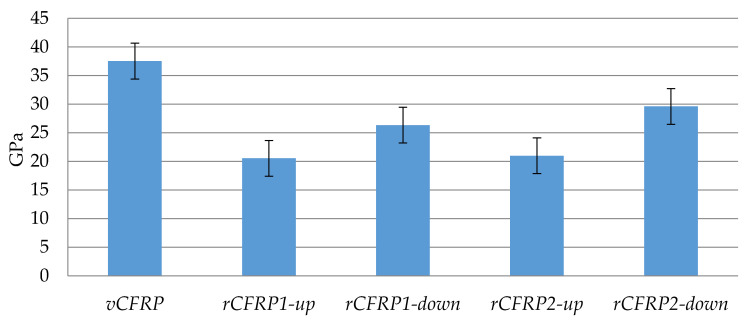
Young modulus of virgin and recycled CFRP pieces.

**Figure 10:**
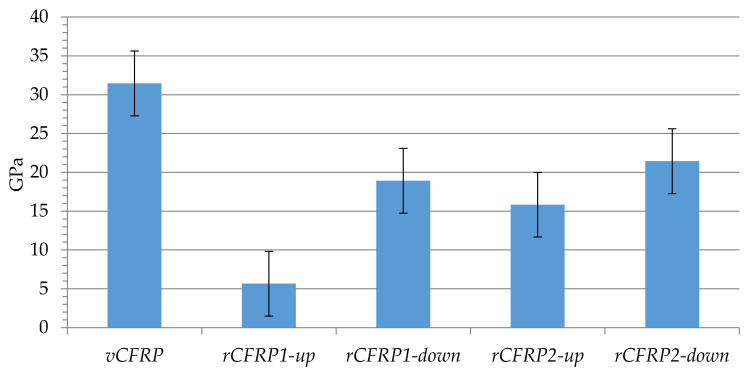
Flexural strength of virgin and recycled CFRP pieces.

**Figure 11:**
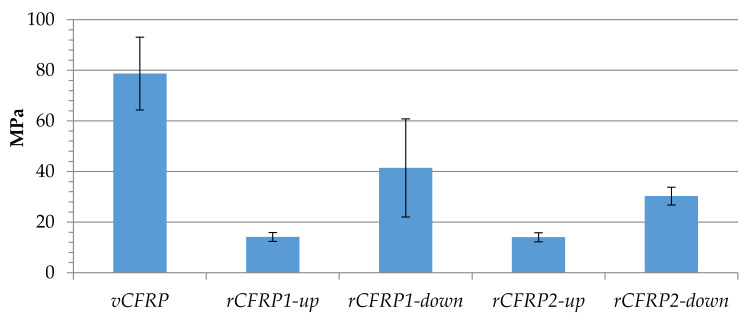
Interlaminar shear strength of virgin and recycled CFRP pieces.

The mechanical properties of the four rCFRP samples are shown in Figure 9, Figure 10 and Figure 11. In all of them, the mean value of the same property but measured in the CFRP pieces fabricated with virgin carbon fiber has been included for comparison, and it is named as vCFRP. It must be taken in consideration that this is not a real piece; vCFRP represents the main value of those properties measured in vCFRP1 and vCFRP2. Besides, it must be said that these results should be analyzed with caution because only two test samples were used per test and type of sample, and there could be sample manufacturing errors affecting the final results. Figure 9 shows the Young modulus of the four rCFRP samples. It can be appreciated that rCFRP pieces were characterized by smaller Young modulus (minimum: 20.5 GPa; maximum: 29.6 GPa) in comparison to the composite material made using virgin carbon fibers (37.5 GPa). In particular, for samples rCFRP1-down and rCFRP1-up, exposed to longer process oxidation times, the value of the Young modulus was slightly smaller (26.3 GPa and 20.5 GPa, respectively) compared to samples rCFRP2-down and rCFRP2-up, coming from O2, i.e., obtained with shorter oxidation times (29.6 GPa and 21.0 GPa, respectively). A longer contact time between air and samples seems to reduce Young modulus, although the rCFRP1 piece contained a slightly higher amount of carbon fiber (see Table 3). Young modulus can be affected by several factors, such as final resin volume content, interphase and intrinsic properties of carbon fibers. Longer oxidation times led to a higher reduction of fiber diameter, as shown by SEM microscopy (Table 6). Damage on the carbon fiber surface and complete removal of sizing probably led to less effective load transfer during tensile test and, consequently, a lower modulus was observed in O1 derived rCFRP parts (rCFRP1-up and rCFRP1-down) compared to those obtained from O2 oxidation (rCFRP2-up and rCFRP2-down). Concerning the fact that the samples located closest to the air feeding point-rCFRP1-down, rCFRP2-down-showed higher Young modulus than those of the upper part of the reactor-rCFRP1-up, rCFRP2-up-this could be explained because the higher amount of char and resin residue surrounding the fibers in the case of materials processed at the upper reactor section limited the infiltration process, leading to poorer interphase and resin accumulation. Both aspects are detrimental for the elastic modulus; the first one by a reduction of load transfer efficiency, the second one by a reduction of effective fiber content which is the responsible for elastic modulus measured through fiber length direction. Flexural strength and interlaminar shear strength will shed light about the hypothesis formulated to describe modulus behavior. In any case, the rCFRP piece presenting the highest Young modulus (rCFRP2-down) showed a reduction of just over 20% with respect to the virgin carbon fiber, which is within the usual range for carbon fibers recovered through pyrolysis and oxidation processes [42].

The flexural strength of the samples can be seen in Figure 10. Again, rCFRP pieces were characterized by smaller flexural strength (minimum: 5.6; maximum: 21.4 GPa) in comparison to the composite material made using virgin carbon fibers (31.5 GPa). Back again, rCFRP1-down and rCFRP1-up (exposed to longer oxidation times) showed the smallest flexural strength values (5.6 GPa and 18.2 GPa, respectively) compared to rCFRP2-down and rCFRP2-up samples (15.8 GPa and 21.4 GPa, respectively), although the rCFRP1 piece contained a slightly higher amount of carbon fiber (see Table 3). Therefore, it could also be said that samples located on the bottom part of the reactor, showing no resin left around the recycled carbon fibers (Figure 4), and showing better adhesion to the new resin used when manufacturing new composite materials. Once again, the sample presenting the best results was rCFRP2-down, with a flexural strength around 68% of that of carbon fiber, similar to results presented by other authors [43].

Figure 11 shows the interlaminar shear strength (ILSS) of virgin and recycled CFRP pieces, revealing significant differences between samples’ values. While the average ILSS value for vCFRP amounted to 78.7 MPa, recycled composite materials broke at stress values smaller than 41.4 MPa. In line with the results obtained in the above presented mechanical tests, samples located closer to the air feeding point in the reactor-rCFRP1-down, rCFRP2-down-showed better resistance values (41.4 and 30.3 MPa, respectively) compared to those located in the upper part-rCFRP1-up, rCFRP2-up-(14.1 MPa and 14.0 MPa, respectively). In addition, samples using recycled carbon fiber fabrics located at the same part (but obtained at different oxidation conditions) showed that, in this case, shorter oxidation times (O2) resulted in smaller values for ILSS: rCFRP1-down (41.4 MPa) vs rCFRP2-down (30.3 MPa), and rCFRP1-up (14.1 MPa) vs rCFRP2-up (14.0 MPa). Nonetheless, the interface between carbon fibers and resin matrix has a major impact on the ILSS, and in this case, the best result barely exceeds 50% of the ILSS value of the original fiber, which is far from some values found in the literature [44]. In view of the results, it seems critical in this case that the carbon fibers were perfectly clean in order to obtain a good ILSS value. Consequently, composite materials made out of carbon fibers recovered in O1 showed higher ILSS values, probably because the carbon fibers surface was perfectly clean after such oxidation conditions, favoring carbon fiber wettability. ILSS is mainly dominated by the adhesion properties between fiber and resin, which can be promoted by surface oxidation due to morphological changes on carbon fiber surface and to the incorporation of oxygen rich functional groups that can improve the interaction between the resin and the fiber [45]. Nevertheless, the effect on the fiber can suppose a reduction of the fiber strength, which is responsible of the decrease on the other mechanical properties observed before. Consequently, the final process must meet a compromise between the enhancement on the matrix-fiber interphase and the decrease of the fiber intrinsic mechanical properties, particularly, their strength due to their high sensitivity for structural fiber damage that leads to its premature failure. 

## 4. Conclusions

The author’s patented method for treating vapors and gases coming from the decomposition of waste CFRP has been successfully applied for the first time to cured CFRP pieces. The results presented in this article show that this method can be applied to epoxy cured samples, and that the properties of the gases and liquids generated are of similar or even better quality than those obtained with uncured epoxy-based CFRP residues. Specifically, gaseous fractions exclusively composed of H_2_, CH_4_ and CO, and with a percentage of H_2_ higher than 66% by volume, have been obtained. At the same time, if the treatment takes place at high temperatures (900 °C), a minority aqueous liquid fraction with very low content of phenol and derivatives is produced, in contrast to the complex organic liquids that are usually obtained in waste CFRP pyrolysis. In terms of matters to be improved, it would be possible to use a catalytic treatment to produce a liquid with a lower organic load than that obtained in the experiments presented in this work. Concerning the reclamation of carbon fibers, reclaimed carbon fiber fabrics have been produced, maintaining the shape and structure of the initial material without the need of further processing. This is a very interesting result, as many recycling approaches obtain fluffy products or separate fibers that must be again treated or used as mats or other reinforcements configurations. It has been observed that the properties of the reclaimed carbon fibers and the composites made with reclaimed carbon fibers are highly dependent on the conditions of the oxidation stage, not only from the point of view of the operating parameters, but also with regard to contact issues between the air and the fibers themselves. In the best cases, recycled composites with a reduction of 20% in Young modulus and 30% in flexural strength with respect to virgin ones have been obtained, which is in line with the typical values for pyrolysis-reclaimed carbon fibers. However, the loss of interlaminar shear strength in the best sample is almost 50%. In this case, contrary to Young modulus and flexural strength, it seems that the fiber is required to be completely clean and partially oxidized to get better results. Based on the results obtained from the mechanical tests, it could be said that optimal carbon fiber recycling process conditions will depend on the new composite material application, since the properties of the recycled fibers and CFRP are highly dependent on the conditions of the oxidation process. In this sense, it is necessary to better control the oxidation process, in order to obtain fabrics that are homogeneously oxidized, without differences between the upper and lower parts. Nevertheless, it could be concluded that the recycling process for cured epoxy composite materials presented in this article is technically feasible, and can produce, at the same time, recycled carbon fiber fabrics and hydrogen-rich gases.

## Figures and Tables

**Table 1:** Main mechanical properties of the carbon fiber fabric.

Carbon Fiber	Tensile Strength (MPa)	Tensile Modulus (GPa)	Elongation at Break (%)	Density (g cm^−3^)
AS4C 3K	4385	231	1.8	1.78

**Table 2:** Main properties of precursor, hardener and their mixture to make the epoxy system.

Component	Viscosity (25 °C) (mPa·s)	Density (g cm^−3^)	Gel Time (140 °C) (Min)
Araldite® LY556	10,000–12,000	1.15–1.2	−
Araldite® XB3473	95–145	0.99–1.02	−
LY556/XB3473 (100/23 wt.%)	6000	1.12–1.16	35–43

**Table 3:** Main properties of virgin and recycled CFRP pieces.

Sample Name	vCFRP1	vCFRP2	rCFRP1	rCFRP2
Dimensions (cm)	13 × 20	13 × 20	13 × 20	13 × 20
Fiber content (wt. %)	65.8	67.6	67.3	65.3
Fiber content (vol. %)	57.7	59.9	58.8	56.6

**Table 4:** Experimental conditions of the carbon fiber recovery processes.

Initial Sample	vCFRP1	vCFRP2
**Pyrolysis step**	**P1**	**P2**
Tank reactor final temperature (°C)	500	500
Tank reactor heating rate (°C min^−1^)	3	3
Tubular reactor temperature (°C)	700	900
**Oxidation step**	**O1**	**O2**
Mass of sample after pyrolysis (g)	77.1	79.8
Mass to be burned (g)	11.3	12.2
T (°C)	500	500
t (min)	120	30
Air flowrate (NL min^−1^)	1.9	4.0
Air excess (%)	40	0

**Table 5:** Yields and composition of products obtained after the recovery process.

Sample	vCFRP1	vCFRP2
Pyrolysis Step	P1 (500/700)	P2 (500/900)
**Pyrolysis yields**	**wt. %**	
Solid	72.6	75.9
Condensates	23.4	14.2
*Collected liquids*	*8.9*	*1.7*
*Other condensates*	*14.5*	*12.5*
Gases ^1^	4.0	9.9
**Pyrolysis gas composition**	**vol. %**	
H_2_	81.7	66.4
CO	15.5	18.0
CO_2_	<0.1	<0.1
CH_4_	2.8	15.6
*HHV (MJ Nm^−3^)*	*12.4*	*15.5*
**Pyrolysis liquids’ composition**	**% area**	
Water	2.3	81.4
Phenol	50.3	6.8
Phenol derivatives	21.0	2.5
Aromatic hydrocarbons	16.0	n.d. ^2^
Other identified compounds	5.3	n.d. ^2^
Not identified	5.1	9.3
**Oxidation step**	**O1 (120/1.9/40)**	**O2 (30/4/0)**
**Remaining solid after oxidation (wt. %) ^3^**	**54.7**	**69.3**

^1^ By difference, ^2^ Not detected, ^3^ Remaining solid with respect to the initial vCFRP mass.

**Table 6:** Average cross-sectional diameter for the virgin carbon fibers (vCF) sample, the recovered carbon fibers in O1 experiment located on the bottom part of the reactor (rCF1-down) sample and the recovered carbon fibers in O2 experiment located on the bottom part of the reactor (rCF2-down) sample.

Sample	Average Cross-Sectional Diameter (µm)
vCF	7.25 ± 0.34
rCF1-down	7.06 ± 0.14
rCF2-down	7.15 ± 0.19

**Table 7:** Full Width at Half Maximum (FWHM) and the value of the diffraction plane (2θ) trhough X-ray diffraction analysis of virgin carbon fibers (vCF) and recovered carbon fibers in O1 experiment (rCF1-down and rCF1-up) and O2 experiment (rCF1-down and rCF1-up).

Sample	FWHM	2θ
vCF	4.6754	25.5301
rCF1-down	4.09411	25.9785
rCF1-up	4.1091	25.7940
rCF2-down	4.3956	25.8036
rCF2-up	4.5776	25.5824

## Data Availability

Not applicable.

## References

[1] Hughes A. (2021). Building and Constructions Plastic Market—An Analysis. Reinf. Plast..

[2] Francis S. (2019). The State of Recycled Carbon Fiber. Compos. World..

[3] Mazumdar S., Pichler D., GangaRao H., Benevento M., Liang R., Witten E. (2019). Industry Report. Materials and Markets in Composites Industry. Compos. Manuf..

[4] Moyer K., Meng C., Marshall B., Assal O., Eaves J., Perez D., Karkkainen R., Roberson L., Pint C.L. (2020). Carbon fiber reinforced structural lithium-ion battery composite: Multifunctional power integration for CubeSats. Energy Storage Mater..

[5] Javaid A., Khalid O., Shakeel A., Noreen S. (2021). Multifunctional structural supercapacitors based on polyaniline deposited carbon fiber reinforced epoxy composites. J. Energy Storage.

[6] van de Werken N., Tekinalp H., Khanbolouki P., Ozcan S., Williams A., Tehrani M. (2020). Additively manufactured carbon fiber-reinforced composites: State of the art and perspective. Addit. Manuf..

[7] Zhu S., Shi R., Qu M., Zhou J., Ye C., Zhang L., Cao H., Ge D., Chen Q. (2021). Simultaneously improved mechanical and electromagnetic interference shielding properties of carbon fiber fabrics/epoxy composites via interface engineering. Compos. Sci. Technol..

[8] Hiremath N., Young S., Ghossein H., Penumadu D., Vaidya U., Theodore M. (2020). Low cost textile-grade carbon-fiber epoxy composites for automotive and wind energy applications. Compos. Part B Eng..

[9] Si H., Zhou L., Wu Y., Song L., Kang M., Zhao X., Chen M. (2020). Rapidly reprocessable, degradable epoxy vitrimer and recyclable carbon fiber reinforced thermoset composites relied on high contents of exchangeable aromatic disulfide crosslinks. Compos. Part B Eng..

[10] Black S. (2017). Composites recycling: Gaining traction. Compos. World.

[11] Holmes M. (2018). Recycled carbon fiber composites become a reality. Reinf. Plast..

[12] Hermansson F., Janssen M., Svanström M. (2019). Prospective study of lignin-based and recycled carbon fibers in composites through meta-analysis of life cycle assessments. J. Clean. Prod..

[13] Liu Y., Meng L., Huang Y., Du J. (2004). Recycling of carbon/epoxy composites. J. Appl. Polym. Sci..

[14] Pegoretti A. (2021). Towards sustainable structural composites: A review on the recycling of continuous-fiber-reinforced thermoplastics. Adv. Ind. Eng. Polym. Res..

[15] Karuppannan Gopalraj S., Kärki T. (2020). A review on the recycling of waste carbon fibre/glass fibre-reinforced composites: Fibre recovery, properties and life-cycle analysis. SN Appl. Sci..

[16] Rani M., Choudhary P., Krishnan V., Zafar S. (2021). A review on recycling and reuse methods for carbon fiber/glass fiber composites waste from wind turbine blades. Compos. Part B Eng..

[17] Utekar S., Suriya V.K., More N., Rao A. (2021). Comprehensive study of recycling of thermosetting polymer composites—Driving force, challenges and methods. Compos. Part B Eng..

[18] Pakdel E., Kashi S., Varley R., Wang X. (2021). Recent progress in recycling carbon fibre reinforced composites and dry carbon fibre wastes. Resour. Conserv. Recycl..

[19] Pillain B., Loubet P., Pestalozzi F., Woidasky J., Erriguible A., Aymonier C., Sonnemann G. (2019). Positioning supercritical solvolysis among innovative recycling and current waste management scenarios for carbon fiber reinforced plastics thanks to comparative life cycle assessment. J. Supercrit. Fluids.

[20] He D., Soo V.K., Kim H.C., Compston P., Doolan M. (2020). Comparative life cycle energy analysis of carbon fibre pre-processing, processing and post-processing recycling methods. Resour. Conserv. Recycl..

[21] Rybicka J., Tiwari A., Leeke G.A. (2016). Technology readiness level assessment of composites recycling technologies. J. Clean. Prod..

[22] Nickels L. (2020). Closing the circle with recycled carbon fiber. Reinf. Plast..

[23] Nickels L. (2019). Smooth sailing with recycled fibers. Reinf. Plast..

[24] Fernández A., Santangelo-Muro M., Fernández-Blázquez J.P., Lopes C.S., Molina-Aldareguia J.M. (2021). Processing and properties of long recycled-carbon-fibre reinforced polypropylene. Compos. Part B Eng..

[25] Liu W., Huang H., Zhu L., Liu Z. (2021). Integrating carbon fiber reclamation and additive manufacturing for recycling CFRP waste. Compos. Part B Eng..

[26] Tapper R.J., Longana M.L., Norton A., Potter K.D., Hamerton I. (2020). An evaluation of life cycle assessment and its application to the closed-loop recycling of carbon fibre reinforced polymers. Compos. Part B Eng..

[27] Lopez-Urionabarrenechea A., Gastelu N., Acha E., Caballero B.M., Orue A., Jiménez-Suárez A., Prolongo S.G., de Marco I. (2020). Reclamation of carbon fibers and added-value gases in a pyrolysis-based composites recycling process. J. Clean. Prod..

[28] Lopez-Urionabarrenechea A., Gastelu N., Acha E., Caballero B.M., de Marco I. (2021). Production of hydrogen-rich gases in the recycling process of residual carbon fiber reinforced polymers by pyrolysis. Waste Manag..

[29] Gastelu N., Lopez-Urionabarrenechea A., Acha E., Caballero B.M., de Marco I. (2019). Evaluation of HZSM-5 Zeolite as Cracking Catalyst for Upgrading the Vapours Generated in the Pyrolysis of an Epoxy-Carbon Fibre Waste Composite. Top. Catal..

[30] Gastelu N., Lopez-Urionabarrenechea A., Solar J., Acha E., Caballero B.M., López F.A., de Marco I. (2018). Thermo-Catalytic Treatment of Vapors in the Recycling Process of Carbon Fiber-Poly (Benzoxazine) Composite Waste by Pyrolysis. Catalysts.

[31] Lopez-Urionabarrenechea A., de Marco I., Caballero B.M., Gastelu N., Hernández A., Adrados A., Solar J. (2016). Method for Treating Vapours Generated during the Process for Recovering Carbon Fibres from Composites by Pyrolysis. WO/2016/135359. https://patentscope.wipo.int/search/en/detail.jsf?docId=EP209578786&tab=NATIONALBIBLIO.

[32] Irisawa T., Aratake R., Hanai M., Sugimoto Y., Tanabe Y. (2021). Elucidation of damage factors to recycled carbon fibers recovered from CFRPs by pyrolysis for finding optimal recovery conditions. Compos. Part B Eng..

[33] Tranchard P., Duquesne S., Samyn F., Estèbe B., Bourbigot S. (2017). Kinetic analysis of the thermal decomposition of a carbon fibre-reinforced epoxy resin laminate. J. Anal. Appl. Pyrolysis.

[34] Mazzocchetti L., Benelli T., D’Angelo E., Leonardi C., Zattini G., Giorgini L. (2018). Validation of carbon fibers recycling by pyro-gasification: The influence of oxidation conditions to obtain clean fibers and promote fiber/matrix adhesion in epoxy composites. Compos. Part A Appl. Sci. Manuf..

[35] Lopez-Urionabarrenechea A., Acha E., Adrados A., Solar J., Caballero B.M., de Marco I. (2020). Use of a reforming catalyst for hydrogen production in the carbonization process of torrefied biomass. Catalysts.

[36] Jiang J., Deng G., Chen X., Gao X., Guo Q., Xu C., Zhou L. (2017). On the successful chemical recycling of carbon fiber/epoxy resin composites under the mild condition. Compos. Sci. Technol..

[37] Qazi H., Lin R., Jayaraman K. (2021). Fibre structure preservation in composite recycling using thermolysis process. Resour. Conserv. Recycl..

[38] Abdou T.R., Botelho Junior A.B., Espinosa D.C.R., Tenório J.A.S. (2021). Recycling of polymeric composites from industrial waste by pyrolysis: Deep evaluation for carbon fibers reuse. Waste Manag..

[39] López F.A., Rodríguez O., Alguacil F.J., García-Díaz I., Centeno T.A., García-Fierro J.L., González C. (2013). Recovery of carbon fibres by the thermolysis and gasification of waste prepreg. J. Anal. Appl. Pyrolysis.

[40] Giorgini L., Benelli T., Mazzocchetti L., Leonardi C., Zattini G., Minak G., Dolcini E., Cavazzoni M., Montanari I., Tosi C. (2015). Recovery of carbon fibers from cured and uncured carbon fiber reinforced composites wastes and their use as feedstock for a new composite production. Polym. Compos..

[41] Park J.M., Kwon D.J., Wang Z.J., Gu G.Y., Devries K.L. (2013). Effect of thermal treatment temperatures on the reinforcing and interfacial properties of recycled carbon fiber-phenolic composites. Compos. Part A Appl. Sci. Manuf..

[42] Yang J., Liu J., Liu W., Wang J., Tang T. (2015). Recycling of carbon fibre reinforced epoxy resin composites under various oxygen concentrations in nitrogen–oxygen atmosphere. J. Anal. Appl. Pyrolysis.

[43] Onwudili J.A., Miskolczi N., Nagy T., Lipóczi G. (2016). Recovery of glass fibre and carbon fibres from reinforced thermosets by batch pyrolysis and investigation of fibre re-using as reinforcement in LDPE matrix. Compos. Part B Eng..

[44] Baek Y.M., Shin P.S., Kim J.H., Park H.S., Kwon D.J., DeVries K.L., Park J.M. (2018). Investigation of interfacial and mechanical properties of various thermally-recycled carbon fibers/recycled PET composites. Fibers Polym..

[45] Li Z., Wu S., Zhao Z., Xu L. (2014). Influence of surface properties on the interfacial adhesion in carbon fiber/epoxy composites. Surf. Interface Anal..

